# Etymologia: Pertactin

**DOI:** 10.3201/eid2004.ET2004

**Published:** 2014-04

**Keywords:** etymologia, pertactin, Bordetella pertussis, bacteria, virulence factor, adhesion, tracheal epithelial cells, neutrophil-mediated clearance, pertussis vaccines

## Pertactin [per-takʹtin]

From *per-* (pertussis) + *tactus* (Latin, “to touch”), pertactin is a virulence factor of *Bordetella pertussis* that promotes adhesion to tracheal epithelial cells and resistance to neutrophil-mediated clearance and is a component of acellular pertussis vaccines. Pertactin-negative *B. pertussis* has been reported in several countries, and its prevalence in the United States has increased in recent years. However, evidence suggests that other components of current pertussis vaccines provide protection against pertactin-negative strains.
